# Three-Dimensional Mandibular Condyle Remodeling Post-Orthognathic Surgery: A Systematic Review

**DOI:** 10.3390/medicina60101683

**Published:** 2024-10-14

**Authors:** Zygimantas Petronis, Audra Janovskiene, Jan Pavel Rokicki, Dainius Razukevicius

**Affiliations:** Department of Maxillofacial Surgery, Lithuanian University of Health Sciences, Eiveniu Str. 2, 44307 Kaunas, Lithuania; audra.janovskiene@stud.lsmu.lt (A.J.); dainius.razukevicius@lsmu.lt (D.R.)

**Keywords:** temporomandibular joint, orthognathic surgery, osteotomies, condyle, mandibular advancement, mandibular setback

## Abstract

*Background and Objectives:* The most popular surgical procedures among orthognathic surgeries for Class II and III patients are Le Fort 1 osteotomy for the maxilla and bilateral sagittal split ramus osteotomy (BSSRO) for the mandible. Keeping the condyle in its proper place during fixation is one of the difficulties of orthognathic surgery. One of the worst post-orthognathic surgery consequences in the temporomandibular joint (TMJ) area may be condylar resorption. Condylar remodeling refers to a group of processes that occur in reaction to forces and stress placed on the temporomandibular joint in order to preserve morphological, functional, and occlusal balance. A systematic review of the literature was performed with the aim of identifying the mandibular condylar component of TMJ changes after orthognathic surgery in class II and III patients. *Materials and Methods:* An electronic search was carried out using the PubMed, Cochrane Library, and Google Scholar, databases. The inclusion criteria included trials in non-growing patients upon whom orthognathic surgery was performed due to Angle II or Angle III classes malocclusion; in addition, a CT or cone beam computed tomography (CBCT) scan was performed before and after surgery to track the mandibular condylar component of TMJ changes. The quality of the studies was evaluated by two independent authors. The risk of bias was assessed by using the Downs and Black checklist. *Results:* The electronic and manual literature search yielded 12 studies that fulfilled all necessary inclusion criteria. Observed studies were evaluated as good (3), fair (8), and poor (1) quality. Two studies evaluated class II patients, six studies observed class III patients, and four studies were comparative. Most of the studies evaluated condyle angle and space changes, and the condylar surface and volume changes were also observed. However, the methodology of evaluation in the publications differs. *Conclusions:* Reduction of bone density, especially in class II patients, and morphological condyle reshaping, with the apposition of the bone, is the main adaptive mechanism after orthognathic surgery. However, all of the studies we examined were conducted using different methods of evaluation, measurement, and reference points.

## 1. Introduction

Orthognathic abnormalities have been treated surgically using a variety of techniques [1]. The most popular surgical procedures among these are Le Fort 1 osteotomy for the maxilla and bilateral sagittal split ramus osteotomy (BSSRO) for the mandible [2]. Although both approaches are often used separately, mandibular prognathism combined with maxillary retrognathism or vice versa treatment requires combining these two methods [1].

Keeping the condyle in its proper place during fixation is one of the difficulties of orthognathic surgery [3,4]. One of the worst post-orthognathic surgery consequences in the temporomandibular joint (TMJ) area may be condylar resorption [5]. Condylar remodeling refers to a group of processes that occur in reaction to forces and stress placed on the temporomandibular joint in order to preserve morphological, functional, and occlusal balance [6].

The post-operative condylar position is believed to be related to surgical stability and post-operative morphological alterations in the TMJ [4]. In published studies, it has been reported that post-operative stability following orthognathic surgery may also be correlated with skeletal angle class II condylar resorption [7]. The patient’s posture during surgery, the tensional balance of the nearby muscles, an insufficient rigid fixation, edema or intracapsular bleeding, asymmetrical surgical movement, or a combination of these factors can result in condyle position changes during orthognathic surgery [3,8]. Other factors may also have an impact, including the distal segment’s direction and amount of movement, the proximal segment’s anatomical shape and orientation, and the surgeon’s experience [8,9,10].

Although the majority of surgical procedures are thought to be very stable, especially in single-jaw procedures, other complications may arise in addition to post-operative relapse [11]. Incorrect condylar positioning can also cause anterior open bites, temporomandibular disorders (TMD), and idiopathic condylar resorption [12]. In addition to ensuring stability, and maintaining the condyles in the proper position, it is crucial to avoid unsatisfactory surgical outcomes [13].

Between skeletal class II and class III populations, there can be significant differences in the type of susceptibility to condylar resorption following orthognathic surgery. This is in contrast to patients with class I malocclusion, who have significant variances in joint space, condyle dimensions, and joint fossa morphology [14].

The effect of the treatment method on condylar remodeling and the potential of condylar resorption aggravation should be highlighted by studying long-term condylar remodeling in the same patients, both during pre-surgical orthodontic treatment and after orthognathic surgery [15]. Currently, and compared to traditional radiography, computed tomography (CT) and three-dimensional tools provide an acceptable technique with excellent accuracy to examine skeletal alterations and condylar remodeling [16].

A lack of consensus exists about the approaches’ accuracy for evaluating changes in the condylar position [17]. It is challenging to compare data from various studies and evaluate the efficacy of new treatments because there is no standardized approach to measure post-operative changes [18].

A systematic review of the literature was performed with the aim of identifying the mandibular condylar component of TMJ changes after orthognathic surgery in class II and III patients.

## 2. Materials and Methods

We conducted this study according to the Preferred Reporting Items for Systematic Reviews and Meta-Analyses (PRISMA) guidelines [19]. The following PICO strategy was established:

The participants (P)—Non-growing Class II and III patients who decided to undergo orthognathic surgery

The intervention (I)—orthognathic surgery

The comparison (C)—class II and III patients

The outcomes (O)—3-dimensional changes of the condyle

The protocol for this systematic review and meta-analysis was registered as PROSPERO CRD42023390032.

### 2.1. Search Strategy

An electronic search was carried out using the PubMed, Cochrane Library, and Google Scholar, databases.

The following keywords were used in the search strategy: (‘temporomandibular joint’ OR ‘tmj’) and (‘orthognathic surgery’ OR ‘osteotomies’).

The literature search was restricted to articles written in the English language and published within the past 5 years, from January 2019 to April 2024. No search limitations concerning publication country or status were applied.

The titles and abstracts were reviewed by two authors independently, who decided whether they met inclusion or exclusion criteria. When the abstracts and titles were cross-checked, the authors independently reviewed and screened the full text. In the case of disagreement, a third author would make the final decision. The first author extracted necessary data from articles, the second author cross-checked it, and the third author made the final decision.

### 2.2. Selection Criteria

The inclusion criteria included trials in non-growing patients upon whom orthognathic surgery was performed due to Angle II or Angle III classes malocclusion; in addition, a CT or cone beam computed tomography (CBCT) scan was performed before and after surgery to track the mandibular condylar component of TMJ changes. Other inclusion criteria were (1) Randomized controlled trials (RCTs); (2) Non-randomized controlled clinical trials; (3) Observational cohort studies; (4) Cross-sectional studies.

In the study, trials where orthognathic surgery was performed due to asymmetry, open front bite, or TMJ disorder were excluded. Furthermore, case reports, series, literature reviews, and meta-analyses were excluded.

### 2.3. Risk of Bias

The quality of the studies was evaluated by two independent authors. The risk of bias was assessed by using the Downs and Black checklist [20]. A total of 27 questions in the checklist were evaluated as 0 or 1 scores, where 0 is not meeting the criteria, and 1 is meeting the criteria. If less than 14 criteria were met by the study, the study was evaluated as poor quality; 15–19 was classified as fair, 20–25 as good, and more than 25 as excellent quality. If a disagreement in assessment appeared, a third author was consulted in order for a consensus to be achieved.

## 3. Results

### 3.1. Study Selection

**Figure 1:**
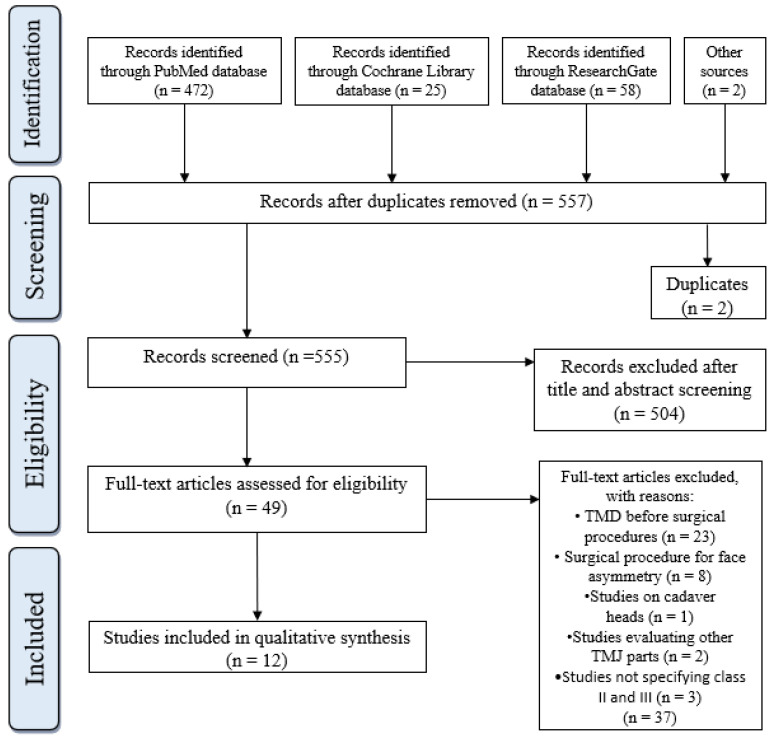
Flow diagram of included searches.

The electronic and manual literature search yielded 557 articles, of which 2 were duplicates and excluded. A total of 555 articles were included in the title and abstract screening. After the eligibility process, 49 records were obtained and the full text of the related studies was read A total of 12 articles fulfilled all necessary inclusion criteria in this systematic review (Figure 1) [21,22,23,24,25,26,27,28,29,30,31,32].

### 3.2. Quality Assessment of the Included Studies

The full Downs and Black checklist was applied for the assessment. The included studies were evaluated as good (3) [22,25,28], fair (8) [21,24,26,27,29,30,31,32], and poor (1) [23] quality and, as a result of the checklist, criteria scores ranged from 14 to 21 points. The aim of the study, the characteristics of included and excluded patients, and the time period during which patients agreed to participate were clearly described in the majority of studies. Furthermore, when considering different studies, all of the patients were recruited from the same population. Patients in these studies cannot be randomized or blinded regarding specific interventions. Moreover, two studies gave a clear description of patients that were lost to follow-up. Criteria for scoring and signaling questions are described in Table 1.

**Table 1:** The Downs and Black checklist.

		Al-Rezami et al., 2022 [21]	Claus J. D.P. et al., 2019 [22]	Kazemian M. et al., 2022 [23]	Lee Ch. et al., 2022 [24]	Wang C. et al., 2021 [25]	Ma R. et al., 2019 [26]	Kucukcakir O. et al., 2022 [27]	Kim J.Y. et al., 2024 [28]	Raluca R et al., 2022 [29]	Abbate et al., 2022 [30]	Hsu L. et al., 2021 [31]	Ueki et al., 2020 [32]
REPORTING													
1. Is the objective of the study clear?	Yes = 1, No = 0	1	1	1	1	1	1	1	1	1	1	1	1
2. Are the main outcomes clearly described in the Introduction or Methods?	Yes = 1, No = 0	0	1	0	1	0	0	0	1	0	0	1	0
3. Are characteristics of the patients included in the study clearly described?	Yes = 1, No = 0	1	1	1	0	1	0	1	0	1	1	1	0
4. Are the interventions clearly described?	Yes = 1, No = 0	1	1	0	1	1	1	1	1	1	1	1	1
5. Are the distributions of principal confounders in each group of subjects clearly described?	Yes = 2, Partially = 1, No = 0	0	0	0	0	1	0	0	1	1	1	0	0
6. Are the main findings of the study clearly described?	Yes = 1, No = 0	1	1	0	1	1	1	1	1	1	1	1	1
7. Does the study estimate random variability in data for main outcomes?	Yes = 1, No = 0	1	1	1	0	1	0	0	1	1	1	1	0
8. Have all the important adverse events consequential to the intervention been reported?	Yes = 1, No = 0	0	0	0	0	0	0	0	0	0	0	0	0
9. Have characteristics of patients lost to follow-up been described?	Yes = 1, No = 0	0	1	0	0	0	0	1	0	0	0	0	0
10. Have actual probability values been reported for the main outcomes except probability < 0.001?	Yes = 1, No = 0	1	0	0	0	1	0	1	1	1	1	1	1
EXTERNAL VALIDITY													
11. Were subjects who were asked to participate in the study representative of the entire population recruited?	Yes = 1, No = 0, Unclear = 0	0	0	0	0	0	0	0	0	0	0	0	0
12. Were those subjects who were prepared to participate representative of the recruited population?	Yes = 1, No = 0, Unclear = 0	0	0	0	0	0	0	0	0	0	0	0	0
13. Were staff, places, and facilities where patients were treated representative of treatment most received?	Yes = 1, No = 0, Unclear = 0	0	0	0	0	0	0	0	1	0	0	0	0
INTERNAL VALIDITY													
14. Was an attempt made to blind study subjects to the intervention?	Yes = 1, No = 0, Unclear = 0	0	0	0	0	0	0	0	0	0	0	0	0
15. Was an attempt made to blind those measuring the main outcomes?	Yes = 1, No = 0, Unclear = 0	0	0	0	0	0	0	0	0	0	0	0	0
16. If any of the results of the study were based on data dredging was this made clear?	Yes = 1, No = 0, Unclear = 0	1	1	1	1	1	0	1	0	0	0	0	0
17. Was the time period between intervention and outcome the same for intervention and control groups or adjusted for?	Yes = 1, No = 0, Unclear = 0	0	1	1	1	1	1	1	1	1	1	1	1
18. Were the statistical tests used to assess main outcomes appropriate?	Yes = 1, No = 0, Unclear = 0	1	1	1	1	1	1	1	1	1	1	1	1
19. Was compliance with the interventions reliable?	Yes = 1, No = 0, Unclear = 0	1	1	1	1	1	1	1	1	1	1	1	1

### 3.3. Patient Characteristics and Follow-Up Period

In the studies included in this review, all patients evaluated had fully developed temporomandibular joints (TMJ), with ages ranging from 18 to 54 years [21,22,23,24,25,26,27,28,29,30,31]. This suggests that the sample primarily consisted of adult patients, ensuring that TMJ development was complete at the time of evaluation.

The follow-up periods across the studies varied considerably. Six studies conducted follow-up evaluations 12 months after surgery, allowing for a comprehensive assessment of long-term outcomes [21,22,26,28,30,32]. Three studies performed follow-up at 6 months post-surgery, providing mid-term insights into patient recovery [23,25,27]. Additionally, three studies only assessed patients immediately after surgery, offering short-term postoperative data [24,29,31]. The variability in follow-up duration reflects the different approaches taken to monitor patient outcomes over time.

### 3.4. II Malocclusion

#### 3.4.1. Angle

In the article written by Ueki et al., 2020 [32] statistically significant results were found when evaluating class II patients’ condyles’ angle changes pre- and post-operatively, which measured 32.9° and 30.6°, respectively.

Hsu L. et al. [31] observed changes between pre- and post-operative condylar ramus angle. Exact information about the follow-up time was not available in this article, although the authors indicate that the follow-up scanning was performed after the debonding. The axial ramal angle decreased by 4.18 ± 5.18° with a significant difference being noted (*p* < 0.001).

#### 3.4.2. Joint Space

In the article by Hsu L. et al. [31], it was reported that there were significant post-operative changes in the anterior and posterior joint space dimensions in class II malocclusion patients (*p* < 0.05) (2.45 to 2.87 mm anterior, and 2.75 to 3.49 mm posterior).

#### 3.4.3. Condylar Surface

Al-Rezami K.F. et al. [21] and Claus J.D.P. et al. [22] evaluated minimum and maximum changes of the condylar surface before and after surgery (15.5 ± 5.5 and 12–19 months respectively). Al-Rezami K.F. et al. [21] observed that the changes ranged from −3.1 at the superior surface to a maximum of 2.9 at the medial surface post-operatively. Claus J.D.P. et al. [22] also noted that the highest resorption was observed in the superior surface (−0.55), and the maximum amount of apposition was in the anterior surface (0.51).

#### 3.4.4. Condylar Volume

In the article by Al-Rezami K.F. et al. [21], there was 12.2% condylar volume in the post-surgical phase (mean 15.5 ± 5.5 months), and the results of this study were statistically significant (*p* = 0.001).

### 3.5. III Malocclusion

#### 3.5.1. Angle

Analyzing mandibular condyle angle changes, Kazemian M. et al. [23] noticed a bilateral decrease in the condyle axial angle after orthognathic surgery; however, results in numbers were not provided in this article.

One article could not be compared with the others, because Lee Y.C. et al. [24] used the y, x, and z-axis for evaluation. On the y-axis, the changes in condylar position were mainly observed downwards (−1.09 ± 0.62 mm) (*p* < 0.05). The changes in the x-axis (0.02 ± 0.68 mm) and z-axis (0.01 ± 0.48 mm) showed no significant differences between before and after orthognathic surgery.

Ma R. et al. [26] found significant differences in mediolateral, craniocaudal translational, axial, sagittal, and coronal angular measurements when comparing follow-up intervals, not pre- and post-surgical results. The condyles moved anteriorly by 0.21 mm and the translational changes in coronal and sagittal view were <0.1 mm on average, 1 year after the operation.

In the article written by Raluca R. et al. [29], condyle angles pre- and post- surgery were 16.34 ± 5.91 and 18.17 ± 5.52 (*p* < 0.05), respectively.

There were two articles that compared intercondylar angles. Wang C. et al. [25] measured pre- and post-surgical results (161.61 ± 5.08 and 159.28 ± 4.92, *p* = 0.061). Abbate et al. [30] analyzed variation pre- and post-surgically and identified changes in the right condyle angle, with a mean of 5.37°, and a mean of 1.82° in the left condyle.

In the study by Wang C. et al. [25], the post-surgical position of TM joint condyles presented only a mild change, with the landmarks’ displacement all within a range of 1.2  mm.

#### 3.5.2. Joint Space

Two articles analyzed joint space. Raluca R. et al. [29] measured anterior joint space before and after surgery (2.15 ± 0.63; 2.0 ± 0.62 mm), posterior joint space (2.01 ± 0.44 mm; 2.16 ± 0.80 mm) and medial joint space (2.02 ± 0.72 mm; 2.55 ± 1.11 mm). Statistically significant results were found in measurements of the medial joint space. Kim J.Y. et al. [28] also measured joint space before and after orthognathic surgery. A significant increase was observed (348.29 mm^3^, 31.34%) in the volume of the overall joint space 3 days post-operatively. After 12 months, the total joint space volume increased by 134.54 mm^3^ (12.11%) compared to the results after 3 days (*p* < 0.0001).

### 3.6. Comparison Class II and III

#### 3.6.1. Angle

Four of the included studies observed condylar angle pre- and post-operatively, comparing class II and III malocclusions [29,30,31,32]. One of the included articles, by Abbate et al. [30], showed no significant results in angular changes between two malocclusion classes.

In the article by Raluca R. et al. [29] results showed that in class II, the right condyle ranged between 12.76° and 44.19° pre-surgery and 9.45° to 41.63° post-surgery in the same class. In contrast, class III presented values of the right condyle angulation between 6.58° and 28.71° pre-surgery and 8.02° to 28.40° post-surgery, respectively, which shows bigger changes in the class II patients group. In the article by Hsu L. et al. [31], the change of axial condylar angle for class II patients was 4.18 ± 5.18°, and −1.42 ± 4.38° for class III patients. In the study by Ueki, K. et al. (2020) [31], condylar angles of class II and II patients were divided into upper and lower levels, and the degree was measured. Class II and III had significant differences pre- and postoperatively in the upper level (27.7° and 13.7° respectively), and lower level (30.6° and 22.6°).

#### 3.6.2. Joint Space

Only one of the included articles evaluated joint space pre- and post-operatively for class II and III patients [29]. The joint space was evaluated in the anterior, posterior, and medial sites, and a significant difference was noted in all results of the study (Table 2).

**Table 2:** Joint space pre- and post-operatively for class II and III patients [29].

Article	Change in Class II			Change in Class III		
	Anterior	Posterior	Medial	Anterior	Posterior	Medial
Raluca R. et al., 2022 [29]	0.61	1.16	0.75	0.37	0.43	0.67

## 4. Discussion

Before BSSO, Le Fort I, or any kind of orthognathic surgery is performed, it is necessary to evaluate the correlation between cephalometric and the TMJ, in order to correctly define the skeletal pattern [33]. CBCT 3D imaging is often used to examine intrinsic variability of the mandible in healthy individuals [34]. A number of studies are using CBCT as a tool to obtain the most accurate results when TMJ changes are evaluated [29]. Mandibular condylar remodeling after orthognathic surgery was reported for the first time in the 1970s by Phillips et al. [35]. After that, more and more studies have shown that, post-orthognathic surgery, there are changes in the temporomandibular joint, especially the condyle. When evaluating the changes in the TMJ after orthognathic surgery it has been observed that, after only 6 months, changes in the structure of the joint begin to be detected, and this process can continue for up to two years. Usually, the patient does not feel any symptoms and the reported pain does not signify degeneration of the condyle [36].

The main aim of this study was to identify the mandibular condylar component of TMJ changes after orthognathic surgery. During the review of scientific literature, an analysis of 12 publications was carried out [21,22,23,24,25,26,27,28,29,30,31,32]. Two studies have observed the condylar changes after orthognathic surgeries for angle class II patients [21,22], six studies included angle class III patients [23,24,25,26,27,28], and four studies compared condylar changes for class II and III patients [29,30,31,32]. Angle, joint space, condylar surface, and condylar volume were evaluated when analyzing the studies.

Orthognathic surgery is often associated with mandibular condyle displacement and remodeling. This process is part of TMJ adaptation, which allows the avoidance of massive consequences related to occlusion. However, the capacity to adapt can be exceeded, which leads to the resorption of the condyle, characterized by bone remodeling with a negative effect on the volume of the condyle [37]. Da Silva et al. [38] observed changes in condylar volume and joint space after orthognathic surgery and their results showed that, after six months, the anterior and medial sites of joint space significantly decreased. Raluca et al. in [29] also observed the joint space after orthognathic surgery for class II and class III patients, reporting a strong correlation between the minimal length of the right and left medial joint space.

When considering angular changes of the condyle, Abbate et al. [30] observed the highest changes of all non-comparative studies in this literature review in class III patients, with a mean of 5.37° in the right condyle. A comparative study by Raluca R. et al. [29] showed similar results and confirmed that greater angular changes can be caused for class II patients after orthognathic surgery. Another comparative study by Hsu L. et al. (2021) [31] found that rotations in axial sections increased significantly in the class III patient group, and decreased significantly in the class II group. Similar results were found by Park S.B. et al. [39] and Kim I.Y. et al. [40]. The results of the study by Park S.B. et al. [39] showed that the axial condylar angle rotated inward after Le Fort I osteotomy and mandibular setback with rigid fixation; however, the authors did not indicate whether patients were angle class II or III. Kim I.Y. et al.’s [40] study observed just class II patients, and significant changes in condylar angulations were observed in the double-jaw surgery patients. The authors indicated that the maxilla rotated clockwise as well as the mandible, which caused midfacial deficiencies concomitantly as it moved forward. Because of that, condylar angulations significantly changed in the axial and sagittal views. Kawamata A. et al.’s [41] study showed that, in the class III patient group after BSSO surgery, the mandible was slightly tilted laterally, and after BSRO it was tilted medially; these results show that this could have considerable effects on the coronal ramal angle.

### Study Limitation

A notable limitation of this systematic review is the high degree of heterogeneity among the included studies, particularly in terms of sample populations and methodologies. The studies reviewed involve a wide range of patient groups, with some focusing on class II malocclusion and others on class III, which introduces variability in both the characteristics of the patients and the surgical techniques employed. Furthermore, differing measurement methods and follow-up protocols were used across studies, complicating direct comparisons of outcomes. This variation in study design and patient demographics makes it challenging to draw definitive conclusions or establish standardized guidelines for the post-operative assessment of patients undergoing orthognathic surgery for class II and class III malocclusions.

## 5. Conclusions

The studies evaluated demonstrate that patients without TMJ disease or dysfunction undergo a natural adaptive process of the mandibular condyle. Reduction of bone density in both patient groups, especially in class II patients, and morphological condyle reshaping, with the apposition of the bone, are the main adaptive mechanisms after orthognathic surgery. However, all of the evaluated studies were conducted using different methods of evaluation, measurement, and reference points.

## Data Availability

Data described in the manuscript, code book, and analytic code will be made available upon request, pending application and approval from the corresponding author.

## References

[1] Robinson R.C., Holm R.L. (2010). Orthognathic surgery for patients with maxillofacial deformities. AORN J..

[2] Joachim M.V., Brosh Y., Rivera C.M., Troulis M.J., AbdelRaziq M., Abu El-Naaj I. (2022). Surgical complications of orthognathic surgery. Appl. Sci..

[3] Li J., Ryu S.-Y., Park H.-J., Kook M.-S., Jung S., Han J.J., Oh H.-K. (2017). Changes in condylar position after BSSRO with and without Le Fort I osteotomy via surgery-first approach in mandibular prognathism with facial asymmetry. Oral. Surg. Oral. Med. Oral. Pathol. Oral. Radiol..

[4] Steenen S.A., Becking A.G. (2016). Bad splits in bilateral sagittal split osteotomy: Systematic review of fracture patterns. Int. J. Oral. Maxillofac. Surg..

[5] Wolford L.M., Gonçalves J.R. (2015). Condylar resorption of the temporomandibular joint. Oral. Maxillofac. Surg. Clin. N. Am..

[6] Hwang H.-S., Jiang T., Sun L., Lee K.-M., Oh M.-H., Biao Y., Oh H.-K., Bechtold T.E. (2019). Condylar head remodeling compensating for condylar head displacement by orthognathic surgery. J. Craniomaxillofac. Surg..

[7] Politis C., Van De Vyvere G., Agbaje J.O. (2019). Condylar resorption after orthognathic surgery. J. Craniofac. Surg..

[8] Lee W., Park J.U. (2002). Three-dimensional evaluation of positional change of the condyle after mandibular setback by means of bilateral sagittal split ramus osteotomy. Oral. Surg. Oral. Med. Oral. Pathol. Oral. Radiol. Endod..

[9] Berkoz O., Karaali S., Kozanoglu E., Akalin B.E., Çeri A., Baris S., Marşan G., Cura N., Emekli U. (2020). The relationship between fixation method and early central condylar sagging after bilateral sagittal split ramus osteotomy in orthognathic surgery. J. Craniomaxillofac. Surg..

[10] Wolford L.M., Reiche-Fischel O., Mehra P. (2003). Changes in temporomandibular joint dysfunction after orthognathic surgery. J. Oral. Maxillofac. Surg..

[11] Peleg O., Mahmoud R., Shuster A., Arbel S., Manor Y., Ianculovici C., Kleinman S. (2021). Orthognathic surgery complications: The 10-year experience of a single center. J. Craniomaxillofac. Surg..

[12] Barone S., Cosentini G., Bennardo F., Antonelli A., Giudice A. (2022). Incidence and management of condylar resorption after orthognathic surgery: An overview. Korean J. Orthod..

[13] Steenen S.A., van Wijk A.J., Becking A.G. (2016). Bad splits in bilateral sagittal split osteotomy: Systematic review and meta-analysis of reported risk factors. Int. J. Oral. Maxillofac. Surg..

[14] Class Ii Ding Y.-F., Song L.-J. (2020). Evaluations of the morphology and position of condyle and fossa of adolescent temporomandibular joint with Class II subdivision malocclusions. Shanghai Kou Qiang Yi Xue Shanghai J. Stomatol..

[15] Gulcek B.N., Ozbilen E.O., Biren S. (2023). Changes in the condylar head after orthognathic surgery in Class III patients: A retrospective three-dimensional study. Angle Orthod..

[16] Talmaceanu D., Lenghel L.M., Bolog N., Hedesiu M., Buduru S., Rotar H., Baciut M., Baciut G. (2018). Imaging modalities for temporomandibular joint disorders: An update. Clujul Med..

[17] Gaber R.M., Shaheen E., Falter B., Araya S., Politis C., Swennen G. (2017). A systematic review to uncover a universal protocol for accuracy assess ment of 3-dimensional virtually planned orthognathic surgery. J. Oral. Maxillofac. Surg..

[18] Stokbro K., Aagaard E., Torkov P., Bell R.B., Thygesen T. (2014). Virtual planning in orthognathic surgery. Int. J. Oral. Maxillofac. Surg..

[19] Page M.J., McKenzie J.E., Bossuyt P.M., Boutron I., Hoffmann T.C., Mulrow C.D., Shamseer L., Tetzlaff J.M., Akl E.A., Brennan S.E. (2021). The PRISMA 2020 statement: An updated guideline for reporting systematic reviews. BMJ.

[20] Acuna R. (2018). The feasibility of creating a checklist for the assessment of the methodological quality both of randomised and non-randomised studies of health care interventions (Preprint). JMIR Prepr..

[21] Al-Rezami K.F., Abotaleb B.M., Alkebsi K., Wang R., Al-Nasri A., Sakran K., Aladimi M., Yang P. (2022). Long-term three-dimensional condylar remodeling during presurgical orthodontics and after orthognathic surgery of mandibular retrognathia with high mandibular plane angle. Clin. Oral. Investig..

[22] Claus J.D.P., Koerich L., Weissheimer A., Almeida M.S., Belle de Oliveira R. (2019). Assessment of condylar changes after orthognathic surgery using computed tomography regional superimposition. Int. J. Oral. Maxillofac. Surg..

[23] Kazemian M., Ghadiri Moghaddam N., Anbiaee N., Kermani H., Samiee Rad S. (2022). The clinical and radiographic changes of temporomandibular joint (TMJ) following mandibular set back surgery by bilateral Sagittal Split Osteotomy (BSSO). World J. Plast. Surg..

[24] Lee Y.-C., Sohn H.-B., Park Y.-W., Oh J.-H. (2022). Evaluation of postoperative changes in condylar positions after orthognathic surgery using balanced orthognathic surgery system. Maxillofac. Plast. Reconstr. Surg..

[25] Wang L.-C., Lee Y.-H., Tsai C.-Y., Wu T.-J., Teng Y.-Y., Lai J.-P., Lin S.-S., Chang Y.-J. (2021). Postsurgical stability of temporomandibular joint of skeletal class III patients treated with 2-jaw orthognathic surgery via computer-aided three-dimensional simulation and navigation in orthognathic surgery (CASNOS). Biomed. Res. Int..

[26] Ma R.-H., Li G., Yin S., Sun Y., Li Z.-L., Ma X.-C. (2020). Quantitative assessment of condyle positional changes before and after orthognathic surgery based on fused 3D images from cone beam computed tomography. Clin. Oral. Investig..

[27] Kucukcakir O., Cansiz E., Yey Z., Ozturk M. (2022). Three dimensional evaluation of changes in mandibular condyle position after orthognathic surgery. Res. Square..

[28] Kim J.Y., Yong H.S., Kim T.Y., Kim J.Y., Jeon K.J., Huh J.K. (2024). Volumetric changes in temporomandibular joint space following trans-oral vertical ramus osteotomy in patients with mandibular prognathism: A one-year follow-up study. Sci. Rep..

[29] Raluca R., Almasan O., Hedeșiu M., Baciuț M., Bran S., Popa D., Ban A., Dinu C. (2022). Evaluation of the mandibular condyle morphologic relation before and after orthognathic surgery in class II and III malocclusion patients using cone beam computed tomography. Biology.

[30] Abbate V., Audino G., Dell’Aversana Orabona G., Friscia M., Bonavolonta P., Lo Faro C., Committeri U., Cuéllar C.N., Iaconetta G., Califano L. (2022). Condylar reshape in orthognathic surgery: Morphovolumetric and densitometric analysis based on 3D imaging and digital workflow. J. Maxillofac. Oral. Surg..

[31] Hsu L.-F., Liu Y.-J., Kok S.-H., Chen Y.-J., Chen Y.-J., Chen M.-H., Yao C.-C.J. (2022). Differences of condylar changes after orthognathic surgery among Class II and Class III patients. J. Formos. Med. Assoc..

[32] Ueki K., Moroi A., Takayama A., Yoshizawa K. (2021). Change of lateral pterygoid muscle and temporomandibular disc position after bi-maxillary surgery in class II and III patients. Oral. Maxillofac. Surg..

[33] Almasan O.C., Baciuţ M., Almasan H.A., Bran S., Lascu L., Iancu M., Băciuţ G. (2013). Skeletal pattern in subjects with temporomandibular joint disorders. Arch. Med. Sci..

[34] Coelho J., Armelim Almiro P., Nunes T., Kato R., Garib D., Migueis ACorte-Real A. (2021). Sex and Age Biological Variation of the Mandible in a Portuguese Population- a Forensic and Medico-Legal Approaches with Three-Dimensional Analysis. Sci. Justice.

[35] Phillips R.M., Bell W.H. (1978). Atrophy of mandibular condyles after sagittal ramus split osteotomy: Report of case. J. Oral. Surg..

[36] de Souza Tesch R., Takamori E.R., Menezes K., Carias R.B.V., Dutra C.L.M., de Freitas Aguiar M., Torraca T.S.d.S., Senegaglia A.C., Rebelatto C.L.K., Daga D.R. (2018). Temporomandibular joint regeneration: Proposal of a novel treatment for condylar resorption after orthognathic surgery using transplantation of autologous nasal septum chondrocytes, and the first human case report. Stem Cell Res. Ther..

[37] Ferri J., Nicot R., Maes J.-M., Raoul G., Lauwers L. (2016). Condylar resorptions and orthodontic-surgical treatment: State of the art. Int. Orthod..

[38] da Silva R.J., Valadares Souza C.V., Souza G.A., Ambrosano G.M.B., Freitas D.Q., Sant’Ana E., de Oliveira-Santos C. (2017). Changes in condylar volume and joint spaces after orthognathic surgery. Int. J. Oral. Maxillofac. Surg..

[39] Park S.-B., Yang Y.-M., Kim Y.-I., Cho B.-H., Jung Y.-H., Hwang D.-S. (2012). Effect of bimaxillary surgery on adaptive condylar head remodeling: Metric analysis and image interpretation using cone-beam computed tomography volume superimposition. J. Oral. Maxillofac. Surg..

[40] Kim Y.-J., Oh K.-M., Hong J.-S., Lee J.-H., Kim H.-M., Reyes M., Cevidanes L.H., Park Y.-H. (2012). Do patients treated with bimaxillary surgery have more stable condylar positions than those who have undergone single-jaw surgery?. J. Oral. Maxillofac. Surg..

[41] Kawamata A., Fujishita M., Nagahara K., Kanematu N., Niwa K., Langlais R.P. (1998). Three-dimensional computed tomography evaluation of postsurgical condylar displacement after mandibular osteotomy. Oral. Surg. Oral. Med. Oral. Pathol. Oral. Radiol. Endod..

